# Ethical self-efficacy among healthcare professionals caring for people with dementia: a brief pre- and post-report on the CARE intervention

**DOI:** 10.1186/s12910-024-01106-z

**Published:** 2024-10-09

**Authors:** Frederik Schou-Juul, Lucca-Mathilde Thorup Ferm, Simon Kinch, Sofie Smedegaard Skov, Christian Ritz, Sigurd Lauridsen

**Affiliations:** National Institute of Public Health, University of Southern Denmark, Copenhagen, Denmark

**Keywords:** Dementia care ethics, Ethical decision-making, Healthcare professionals, Ethical self-efficacy

## Abstract

**Background:**

Interventions targeting healthcare professionals’ confidence in managing ethical issues in dementia care are limited despite documented positive effects of educational programs on staff knowledge and self-efficacy. However, inconsistencies in the literature regarding the impact of educational programs underscore the need for targeted interventions. The CARE intervention, specifically designed to enhance confidence in ethical decision-making, aims to address this gap. This study evaluates the effectiveness of the CARE intervention in enhancing the ethical self-efficacy of healthcare professionals caring for people with dementia, particularly those with initially low levels of self-efficacy.

**Methods:**

Using a non-experimental pre-post evaluation design, the CARE intervention was administered to healthcare professionals (*n* = 86), measuring ethical self-efficacy pre-and post-intervention. We hypothesized significant differences in ethical self-efficacy mean scores pre- and post-intervention for all participants, particularly those with low pre-measurement scores, whom we expected to benefit most from the intervention. Statistical analysis included paired t-tests and Wilcoxon tests for the low pre-measurement subgroup analysis.

**Results:**

While no significant change was observed in the entire sample, participants with low initial self-efficacy showed a statistically significant improvement post-intervention.

**Conclusions:**

The CARE intervention holds promise in improving ethical self-efficacy among healthcare professionals with initial low confidence levels. Targeted interventions are essential in addressing confidence gaps in managing ethical challenges in dementia care, with implications for professional well-being and quality of care. Further research should explore long-term effects and expand sample size to enhance generalizability and sustainability of findings.

**Supplementary Information:**

The online version contains supplementary material available at 10.1186/s12910-024-01106-z.

## Introduction

Interventions towards improving healthcare professionals’ confidence in their ability to manage ethical issues in dementia care have been sparse. Educational programs and interventions have been documented as generally having positive, albeit short-term, effects on staff knowledge [1, 2]. Some of these programs and interventions also have significant impacts on the self-efficacy or perceived confidence of healthcare professionals in managing dementia care [3–5]. However, a recent review argues that the effects of educational programs on professionals’ sense of competence or self-efficacy are inconsistent in the literature [2]. This adds to the backdrop of studies showing that a substantial segment of caregivers still lack confidence in managing the challenging behaviour of people with dementia [6, 7].

Various programs have been developed specifically to enhance various domains of dementia care, including programs specifically designed to manage the challenging behaviours of people with dementia [3, 4, 8]. However, to our knowledge, only one intervention has been developed specifically to enhance confidence or self-efficacy in making ethical decisions [9]. Self-efficacy, as defined by Bandura’s theory, refers to an individual’s belief in their ability to perform actions required to achieve specific outcomes [10]. In the context of ethical decision-making in dementia care, self-efficacy reflects healthcare professionals’ confidence in their capacity to handle ethical challenges effectively.

This lack of specific efforts to enhance healthcare professionals’ confidence in addressing ethical issues is notable, especially when considering the high prevalence of ethical issues encountered by healthcare professionals in dementia care [11–13]. Ethical issues in dementia care are well-documented in the literature [14–16], and commonly include, in the early stages, challenges related to autonomy and consent [17], to managing behavioral symptoms of people with dementia in long-term care [18], to making decisions regarding resuscitation and end-of-life issues in the late stages [16]. These issues often require healthcare professionals to navigate conflicting ethical principles while considering the best interests of the person with dementia. The high prevalence of such ethical issues in dementia care is often associated with healthcare professionals experiencing moral distress when navigating these challenges [19, 20]. Building upon this sentiment, studies indicate that confidence in managing ethical challenges may improve professionals’ ability to manage moral distress [21, 22]. It may also potentially reduce their feelings of burnout [23, 24]. The training and education of healthcare professionals has the potential to enhance the well-being of both healthcare professionals and people with dementia [25]. If self-efficacy contributes to better handling of work-related challenges, including ethical dilemmas, and helps to reduce burnout, then interventions designed to boost healthcare professionals’ confidence hold significant promise.

In this article, we are reporting the findings from an evaluation of the CARE intervention, which is aimed specifically at improving healthcare professionals’ confidence in ethical decision-making in dementia care [9]. The aim of this study is to assess whether the CARE intervention successfully enhanced the ethical self-efficacy of healthcare professionals caring for people with dementia. It also places a specific emphasis on improving the ethical self-efficacy of professionals with initial low levels of self-efficacy, as other studies have documented an association between low self-efficacy scores and negative outcomes, including burnout, as such develops when requirements of a given job and workers’ perceived abilities do not match up [26].

## Methods

### Design and hypotheses

The design of this study followed a non-experimental pre-post evaluation design, in which we administered the CARE intervention to healthcare professionals caring for people with dementia with the aim of improving the professionals’ confidence in making ethical decisions [27]. The hypotheses for this study were twofold. First, we expected a significant difference in the mean scores of ethical self-efficacy between the pre-intervention and post-intervention measurements when looking at all participants in these interventions. Second, we anticipated a significant difference in the pre-and post-intervention mean scores among intervention participants who scored low in the pre-measurement. Focusing on individuals with low self-efficacy was based on the assumption that those with lower initial confidence levels are not only at greater risk of negative outcomes associated with low self-efficacy but also have the most potential for improvement from targeted interventions. Therefore, we expected these individuals to benefit most from the intervention.

We measured their confidence using the validated Dementia-Specific Ethical Self-Efficacy (DemESE) scale at pre- and post-intervention periods [28]. Statistical analysis was then conducted to assess how the CARE intervention affected healthcare professionals’ ethical self-efficacy across the entire sample and within the specific subgroup of healthcare professionals who initially had low scores.

### Intervention

The CARE intervention was comprised of two workshop modules following a structured manual, each lasting approximately four hours, with workshops held at 14-day intervals. The intervention was developed by the National Institute of Public Health, University of Southern Denmark, in association with the Danish Alzheimer Association and Rudersdal Municipality.

The intervention was implemented in Rudersdal Municipality and targeted professional caregivers of people with dementia. Recognizing that ethical issues can arise from conflicting ethical principles, CARE was structured in a workshop format, in which healthcare professionals were encouraged to convene and collaboratively address these conflicts [9]. The CARE intervention included workshops specifically designed for healthcare professionals from various long-term care facilities. These workshops aimed to enhance participants’ confidence in managing ethical issues in dementia care through facilitated discussions led by a moderator experienced in dementia care ethics. A distinctive feature was the use of literary cases, such as fictional or autobiographical texts, to present ethical dilemmas in a neutral and engaging manner. Inspired by the traditions of narrative medicine, we hypothesized that incorporating literary texts could enhance the understanding of shared experiences and values in dementia care [29–31]. During the workshops, the facilitator distributed and read aloud excerpts from literary works that depicted challenging situations in dementia care, followed by guided discussion questions. This approach encouraged healthcare professionals to reflect on ethical challenges, fostering empathy and new perspectives by engaging with the scenarios in a way that remained detached from their personal experiences.

The CARE intervention aimed to train caregivers in recognizing, analysing and responding to ethical challenges in dementia care. Throughout the intervention, healthcare professionals were introduced to bioethical theory and the principles of ethical decision-making, which were then discussed in connection with their everyday experiences with ethical decision-making. The underlying idea was that introducing health professionals to ethical principles and having them discuss their everyday experiences of ethical decision-making with their peers would enhance their moral sensitivity, which in turn would bolster their confidence or self-efficacy in addressing ethical issues. For a detailed account of the development of the CARE intervention, please refer to Lauridsen et al. 2023 [9].

### Intervention participants

The participants encompassed a variety of healthcare professionals with varying levels of education and work experience who were working with people with dementia (see Table 1). To assess their eligibility for participation in the intervention, we ensured that the participants worked as caregivers at one of the four participating nursing homes during either day or night shifts, had direct contact with people with dementia and possessed sufficient fluency in Danish to engage in discussions. A total of 113 healthcare professionals participated in the intervention.

**Table 1:** Descriptive statistics

	Entire sample		Low initial score sample	
**Gender**	**N**	**Percent**	**N**	**Percent**
Woman	77	93.9	22	95.7
Man	5	6.1	1	4.3
**Work experience**				
Over 8 years	41	50.0	10	43.5
6–8 years	8	9.8	3	13.0
3–5 years	13	15.9	5	21.7
0–2 years	20	24.4	5	21.7
**Position**				
Social and healthcare helper	32	39.0	8	34.8
Social and health assistant	21	25.6	5	21.7
Untrained worker	7	8.5	3	13.0
Pedagogue	10	12.2	3	13.0
Nurse	6	7.3	3	13.0
Nursing Assistant	2	2.4	0	0
Other	4	4.9	1	4.3
**Continuous variables**	**Mean**	**SD**	**Mean**	**SD**
Age	47.9	11.4	45.8	10.2
DemESE scale score before intervention	28.3	6.8	19.2	3.7
DemESE scale score after intervention	27.4	7.6	21.8	6.0

### Data collection

The intervention participants were recruited through targeted outreach efforts at four different care facilities in Rudersdal Municipality. The recruitment was arranged by a local administrator who coordinated with nursing home managers across the different facilities. These managers, in turn, recruited healthcare professionals from their respective institutions to partake in the intervention by means of convenience sampling. Although convenience sampling was used, efforts were made to select nursing homes with diverse characteristics, such as different sizes, care models, and staff compositions, to ensure that the sample was as representative as possible of the broader range of dementia care settings within the municipality. This sample resembles the overall population of healthcare professionals, as only 3% of Danish nurses and 5% of healthcare assistants are men [32]. We surveyed the intervention participants before and after the intervention, with the facilitator distributing paper surveys just before the commencement of the first module (pre-intervention) and immediately after the conclusion of the final module (post-intervention). The pre-intervention survey included participants’ sociodemographic characteristics and a baseline measurement of their ethical confidence. The post-intervention survey included questions about the intervention’s impact on the respondents’ ethical self-efficacy and their satisfaction with the intervention. The data was collected between December 2021 and November 2022.

### Measurements and variables

We used the validated Dementia-Specific Ethical Self-Efficacy (DemESE) scale to measure our dependent variable, which was healthcare professionals’ perceived confidence in making decisions when there were conflicts between different ethical principles or requirements [28]. The DemESE scale is comprised of six items, and it assesses the confidence levels in ethical dilemmas using a 7-point Likert scale, allowing integer scores between 1 and 7. The DemESE items capture the frequency with which healthcare professionals feel unconfident or uncertain when making ethical decisions. DemESE is comprised of a single scale, ranging from 6 to 42, and a higher total score indicates greater perceived ethical self-efficacy among healthcare professionals. Initial low scores are defined as scores of 0 to 24, corresponding to median or low scores on all questions. This range was chosen because it captures participants whose confidence levels are not consistently high, indicating potential struggles with ethical decision-making, and aligns with our objective of targeting those who are most likely to benefit from the intervention.

### Statistical analysis

Paired t-tests were performed on the entire sample when the assumption of normality was confirmed by the Shapiro-Wilk test and visual inspection of the Q-Q plot. We conducted normality tests for the entire sample at both the pre-and post-intervention phases. The equality of variance was tested with an F-test that confirmed it. These verifications supported the robustness of our data by meeting the necessary assumptions for the subsequent statistical analysis of the entire sample. Since the subsequent analysis focused on the specific subgroup with initial low scores, this subgroup underwent separate normality testing to ensure that we were not violating the assumptions of the statistical test. To account for non-normal distribution in the subgroup analysis, a non-parametric or distribution-free Wilcoxon test was conducted on the intervention participants with initial low scores. We employed R when conducting statistical analysis in this study [33]. A significance level of 0.05 was applied.

### Ethical considerations and funding declarations

This study was part of the DEMENS ID research project, which was approved by the Research Ethics Committee (REC) of the University of Southern Denmark and the legal representatives of the Research & Innovation Organisation (RIO). The intervention participants provided their informed consent prior to taking part in the study. In the introduction to the survey, we clarified that participation in this evaluation was voluntary, emphasizing that non-participation would not result in any consequences nor in any exclusion from participating in the intervention. Participants were fully briefed on the study’s objectives and on the handling of their data.

This study received funding from the Velux Foundation through the HUMPraxis program under Grant Agreement nr. 27,773.

## Results

### Descriptive statistics

In this section, we have presented a detailed overview of the main descriptive statistics of our dataset.

The DemESE scale was included in a larger questionnaire administered to a total of 113 healthcare professionals participating in the intervention. A total of 86 participants completed the baseline survey for this evaluation. Of these, 82 also completed the post-intervention survey, resulting in an overall response rate of approximately 72.6% from the initial 113 eligible participants. The entire sample of this study thus included a total of 82 individuals with a mean age of 47.9 years (SD = 11.4). The participants (see Table 1) had a wide variety of educational backgrounds, as they ranged from untrained workers to nurses and had varying levels of experience. However, there was a propensity towards greater experience, with 41 (50%) of the intervention participants reporting having worked with people with dementia for over eight years. Of the participants, 6.1% were male and 93.9% were female. The low initial score sample included 23 individuals with a mean age of 45.8 years. The proportion of participants with experience of over 8 years was slightly lower (43.5%).

### Test results

We found that the mean score of the entire sample was 28.3 (SD = 6.8) at the pre-intervention stage and 27.4 (SD = 7.6) at the post-intervention stage. Adherence to the normal distribution assumptions was confirmed in both the pre-and post-intervention datasets. There was no statistically significant difference in the means of pre- and post-intervention (*p* = 0.19) in the entire sample. This implies that the CARE intervention seemingly had no significant effect on improving the mean value of ethical self-efficacy throughout the entire sample.

We also found that the intervention subgroup with initial low scores had a mean score of 19.2 pre-intervention (SD = 3.7) and 21.8 (SD = 6.0) post-intervention. We were unable to establish normality in the subgroup analysis of the intervention participants with initial low scores. Among healthcare professionals with initially low self-efficacy levels, there was a statistically significant improvement (*p* = 0.04).

## Discussion

This study was designed to test the hypothesis that the CARE intervention, a specific intervention aimed at enhancing the confidence of healthcare professionals when making ethical decisions, leads to significant enhancements in ethical self-efficacy among healthcare professionals. Although we found that the overall sample did not exhibit a significant change in mean scores post-intervention within the subgroup of participants with initially low self-efficacy, we observed a statistically significant improvement in their confidence levels that was potentially attributable to the CARE intervention.

Building on the existing literature, our study delves into the critical role of self-efficacy in dementia care. The literature has consistently highlighted the perceived difficulty of ethical decision-making and the moral distress associated with ethical dilemmas in dementia care. However, the inconsistency in the literature regarding the impact of educational programs on self-efficacy underscores the need for targeted interventions [3–5]. This inconsistency is notable in light of caregivers continuing to report a lack of confidence in managing the challenging behaviours of people with dementia [6, 7]. In addressing this lack of confidence in managing challenging situations, as reported by certain healthcare professionals in dementia care, the CARE intervention has potential benefits in improving their confidence when making ethical decisions.

While we were unable to document any significant changes in the confidence levels of the complete sample, this may be because our sample of healthcare professionals reported a relatively high level of confidence to begin with (i.e., 28.3 out of a potential maximum of 42). Given the well-documented moral distress among healthcare professionals in dementia care [20], the ethics educational needs in healthcare [34] and the high prevalence of complex ethical issues they regularly encounter [15, 35], it is reasonable to expect that confidence levels would be tempered by these ongoing challenges. It might, therefore, be likely that the frequent ethical dilemmas inherent in dementia care potentially contribute to a somewhat natural ceiling on confidence levels. Alternatively, there may be a decreasing marginal utility associated with the intervention effect as participants approach the potential maximum. However, while we did not expect a high mean for the entire sample, we did expect that we would be unable to make significant changes to the scores of participants with high self-efficacy levels. In fact, we speculated that those with initial high scores would not benefit from the intervention. Their individual self-perceived moral capacity, including their beliefs regarding which ethical decisions were appropriate, may have been challenged during the intervention, thus leading to decreased self-efficacy. Although we cannot assert this is practically the case, the overall sample revealed a minor and statistically insignificant decrease in the participants’ self-efficacy levels.

Exploring the potential mechanisms behind the statistically significant impact of the intervention on the participants who were initially lacking confidence is beyond this paper’s scope. However, it is noteworthy that the elements employed in the CARE intervention align with the approach outlined by Rasmussen, et al. 2023 [36]. They found that interventions in dementia education using classroom teaching contexts combined with practice, behaviour and communication-oriented teaching styles may improve self-efficacy among healthcare professionals. Our study also provides an instance of an intervention in dementia education using person-centred teaching approaches to affect self-efficacy, for which evidence had previously been missing. Our results indicate that such interventions may be able to positively influence self-efficacy in certain healthcare professionals who lack confidence.

### Clinical implications

The findings of this study have important clinical implications for dementia care settings. The CARE intervention’s focus on enhancing ethical self-efficacy among healthcare professionals has the potential to address moral distress and improve decision-making confidence, particularly for those with lower initial self-efficacy. Implementing such targeted interventions in clinical practice can support caregivers in navigating complex ethical dilemmas, thereby contributing to improved care outcomes and the overall well-being of healthcare professionals and potentially also people with dementia if high confidence levels are associated with making better ethical decisions. The results of this study underscore the value of identifying and supporting healthcare professionals who may benefit most from these types of interventions, which can be critical in settings with high ethical demands and challenging care scenarios. Integrating similar programs into routine training and professional development could help build a more resilient and confident workforce capable of handling the ethical complexities inherent in dementia care.

### Limitations

While this study demonstrates robust methodological rigor, it is essential to address certain specific limitations that warrant careful consideration. First, there are potential limitations regarding possible violations of the assumptions within the tests being used. These would include the assumption of equal variance and of normality in the statistical tests. Second, caution should be applied when interpreting the significant effect of the intervention, given the limited number of intervention participants with initially low scores.

This study accounts for potential errors that may arise due to violating these assumptions before the analysis by testing them accordingly. However, several noteworthy limitations persist in the study design. The lack of random assignment is a major weakness of the non-experimental pre-post evaluation. One major challenge is that such studies only have a single arm and, therefore, lack a comparator arm or control group. The associations identified in such studies may be substantiated by an important requirement of causality, such as the intervention occurring before the measurement of the outcome. However, the absence of a control group makes it challenging to establish a causal relationship between the intervention and the observed changes [37]. Notably, among healthcare professionals with initially low self-efficacy levels, a statistically significant improvement was observed. However, in this respect, it is essential to acknowledge the potential influence of regression towards the mean in interpreting this improvement. Caution should be exercised in attributing this small but statistically significant improvement solely to the intervention process.

Notably, another limitation may arise regarding the question of when to follow up and when the measurement of the dependent variable should occur during the post-intervention stage. While we acknowledge that optimal timing depends highly on the nature of the intervention and the expected length of time for the potential effects to manifest, we are also quite confident that our post-measurement was conducted before the effects of the intervention had manifested. Since we measured the post-intervention self-efficacy immediately after the conclusion of the final module of the intervention, the effects of the intervention may not yet have been measurable. However, it is also highly possible that the intervention only exerted a limited measurable effect and that this effect represents the highest achievable measure. This aligns with prior research illustrating the diminishing impact of educational interventions over time [2]. We were also unable to reject our first null hypothesis and found a significant difference in the mean scores of ethical self-efficacy between the pre-intervention and post-intervention measurements throughout the entire sample. However, this is not to say that this group did not gain anything from participating in the intervention.

There may be certain immeasurable effects on intervention participants with high initial scores that are not accounted for in this study. These may include satisfaction with educational elements, reinforcement of beliefs high in self-efficacy, and satisfaction with peer feedback or discussion as methods of maintaining dialogue regarding ethical decision-making when caring for people with dementia. Given the study’s limitations, especially the relatively small number of intervention participants with low initial scores, future research should focus on expanding the sample size within this subgroup. This would enhance the generalizability of the findings and would provide a more comprehensive understanding of the CARE intervention’s impact on ethical self-efficacy. Exploring the long-term effects and conducting follow-up assessments could also offer insights into the sustainability and, potentially, the development of these improvements over time.

## Conclusion

Our findings underscore the importance of targeted interventions, such as the CARE intervention, in addressing confidence gaps among healthcare professionals who are dealing with ethical challenges in dementia care. While the CARE intervention did not enhance the overall confidence of the entire sample, it did have a statistically significant effect on participants with initial low self-efficacy, suggesting that the CARE intervention may be beneficial for this segment of health professionals.

## Electronic supplementary material

Below is the link to the electronic supplementary material.

Supplementary Material 1

## Data Availability

Data and materials are available upon reasonable request. Due to the relative low number of participants in specific demographic groups, there exists a potential risk of deductive disclosure. Therefore, the authors must assess this risk before making the data available. Requests for data and materials should be directed to Frederik Schou-Juul at Fsch@sdu.dk.

## Abbreviations

DemESE scale: Dementia-Specific Ethical Self-Efficacy scale
