# Supplementary material for: Ethical self-efficacy among healthcare professionals caring for people with dementia: a brief pre- and post-report on the CARE intervention
Source: BMC Med Ethics. 2024 Oct 9;25:109. doi: 10.1186/s12910-024-01106-z (PMC11463077; doi:10.1186/s12910-024-01106-z)
Supplement: Supplementary file 1 — Supplementary Material 1 [file 12910_2024_1106_MOESM1_ESM.docx]

# APPENDIX 1: Dementia-Specific Ethical Self-Efficacy (DemESE) scale for professional caregivers (Danish original)

| I det følgende vil vi bede dig besvare en række spørgsmål til hvordan oplever etiske dilemmaer i dit daglige arbejde.  Husk der er ingen rigtige eller forkerte besvarelser! | |
| --- | --- |
| DemESE-item-1 | Hvor ofte er du i tvivl om hvad det rigtige er at gøre, når der er konflikt imellem hvad du synes, er bedst for beboeren og beboerens egne ønsker? |
|  | (Altid) 1 – 2 – 3 – 4 – 5 – 6 – 7 (Aldrig) |
| DemESE-item-2 | Hvor ofte er du i tvivl om hvad det rigtige er at gøre, når der er konflikt imellem hvad du synes, er bedst for beboeren og hvad pårørende ser som en værdig pleje? |
|  | (Altid) 1 – 2 – 3 – 4 – 5 – 6 – 7 (Aldrig) |
| DemESE-item-3 | Hvor ofte er du i tvivl om hvad det rigtige er at gøre, når det som du synes er bedst for den enkelte beboer, samtidig går ud over andre beboere? |
|  | (Altid) 1 – 2 – 3 – 4 – 5 – 6 – 7 (Aldrig) |
| DemESE-item-4 | Hvor ofte er du i tvivl om hvad det rigtige er at gøre, når du synes det som beboeren selv ønsker, er uværdigt? |
|  | (Altid) 1 – 2 – 3 – 4 – 5 – 6 – 7 (Aldrig) |
| DemESE-item-5 | Hvor ofte er du i tvivl om hvad det rigtige er at gøre, når beboerens behov går ud over andre beboere? |
|  | (Altid) 1 – 2 – 3 – 4 – 5 – 6 – 7 (Aldrig) |
| DemESE-item-6 | Hvor ofte er du i tvivl om hvad det rigtige er at gøre, når du skal prioritere mellem hvilke beboeres behov, du skal bruge din tid på? |
|  | (Altid) 1 – 2 – 3 – 4 – 5 – 6 – 7 (Aldrig) |

# APPENDIX 2: Dementia-Specific Ethical Self-Efficacy (DemESE) scale for professional caregivers (English translation)

| In the following, we will ask you to answer a series of questions about how you experience ethical dilemmas in your daily work.  Remember, there are no right or wrong answers! | |
| --- | --- |
| DemESE-item-1 | How often do you have doubts about the right thing to do when there is a conflict between what you think is best for a resident and the resident’s own wishes? |
|  | (Always) 1 – 2 – 3 – 4 – 5 – 6 – 7 (Never) |
| DemESE-item-2 | How often do you have doubts about the right thing to do when there is a conflict between what you think is best for a resident and what their relatives see as dignified care? |
|  | (Always) 1 – 2 – 3 – 4 – 5 – 6 – 7 (Never) |
| DemESE-item-3 | How often do you have doubts about the right thing to do when what you think is best for an individual resident affects other residents? |
|  | (Always) 1 – 2 – 3 – 4 – 5 – 6 – 7 (Never) |
| DemESE-item-4 | How often do you have doubts about the right thing to do when you think that a resident’s wants are undignified? |
|  | (Always) 1 – 2 – 3 – 4 – 5 – 6 – 7 (Never) |
| DemESE-item-5 | How often do you have doubts about the right thing to do when a resident’s needs have consequences for other residents? |
|  | (Always) 1 – 2 – 3 – 4 – 5 – 6 – 7 (Never) |
| DemESE-item-6 | How often do you have doubts about the right thing to do when prioritising which residents’ needs you should spend your time on? |
|  | (Always) 1 – 2 – 3 – 4 – 5 – 6 – 7 (Never) |
